# Antioxidant Activity of *Valeriana fauriei* Protects against Dexamethasone-Induced Muscle Atrophy

**DOI:** 10.1155/2022/3645431

**Published:** 2022-01-12

**Authors:** Young In Kim, Hyunjung Lee, Farida S. Nirmala, Hyo-Deok Seo, Tae Youl Ha, Chang Hwa Jung, Jiyun Ahn

**Affiliations:** ^1^Research Group of Aging Metabolism, Korea Food Research Institute, Wanju, Republic of Korea; ^2^Department of Food Science and Technology, Jeonbuk National University, Jeonju-si, Republic of Korea; ^3^Department of Food Biotechnology, University of Science and Technology, Daejeon, Republic of Korea

## Abstract

Skeletal muscle atrophy is defined as wasting or loss of muscle. Although glucocorticoids (GCs) are well-known anti-inflammatory drugs, their long-term or high-dose use induces skeletal muscle atrophy. *Valeriana fauriei* (VF) is used to treat restlessness, anxiety, and sleep disorders; however, its effects on skeletal muscle health have not been investigated. This study investigated whether *Valeriana fauriei* could ameliorate muscle atrophy. We induced muscle atrophy *in vitro* and *in vivo*, by treatment with dexamethasone (DEX), a synthetic GC. In DEX-induced myotube atrophy, *Valeriana fauriei* treatment increased the fusion index and decreased the expression of muscle atrophic genes such as muscle atrophy F-box (MAFbx/Atrogin-1) and muscle RING-finger protein 1 (MuRF1). In DEX-treated mice with muscle atrophy, *Valeriana fauriei* supplementation increased the ability to exercise, muscle weight, and cross-sectional area, whereas it inhibited myosin heavy chain isoform transition and the expression of muscle atrophy biomarkers. *Valeriana fauriei* treatment led to via the downregulation of muscle atrophic genes via inhibition of GC receptor translocation. *Valeriana fauriei* was also found to act as a reactive oxygen species (ROS) scavenger. Didrovaltrate (DI), an iridoid compound from *Valeriana fauriei*, was found to downregulate atrophic genes and decrease ROS in the DEX-induced myotube atrophy. Consolidated, our results indicate that *Valeriana fauriei* prevents DEX-induced muscle atrophy by inhibiting GC receptor translocation. Further, *Valeriana fauriei* acts as a ROS scavenger, and its functional compound is didrovaltrate. We suggest that *Valeriana fauriei* and its functional compound didrovaltrate possess therapeutic potentials against muscle atrophy.

## 1. Introduction

Skeletal muscle is an important component of the body and is closely related to metabolism and locomotion. However, aging, muscle inactivity (e.g., immobilization or chronic bed rest), denervation, cancer, and injury can trigger loss of muscle mass, leading to an increase in morbidity and mortality [1, 2]. Reduction of muscle mass and muscle function by diverse causes is termed skeletal muscle atrophy [3].

Protein homeostasis, or proteostasis, maintains the balance between protein degradation and protein synthesis. Loss of this homeostasis is associated with protein misfolding and diseases [4]. The loss of homeostasis refers to the imbalance between protein degradation and protein synthesis and causes skeletal muscle loss. Protein degradation is accelerated by the ubiquitin-proteasome system (UPS), a major intracellular proteolysis system [1]. The UPS target protein is ubiquitinated by the action of ubiquitin-activating enzymes (E1), ubiquitin-conjugating enzymes (E2), and ubiquitin ligases (E3). First, E1 activates the ubiquitin-protein and transfers it to one of several ubiquitin-conjugating enzymes, E2s. E2 carries the activated ubiquitin to E3. E3 then specifically conjugates ubiquitin covalently to the substrate, which is a target for degradation. The substrate accumulates poly-ubiquitin, and the ubiquitinated protein is degraded by the 26S proteasome [5]. Muscle atrophy F-box (MAFbx/Atrogin-1) and Muscle RING finger 1 (MuRF1) are muscle-specific E3 ubiquitin ligases that are often used as muscle atrophy biomarkers [6, 7].

Reactive oxygen species (ROS) play important roles in the regulation of physiological processes and are increasingly being shown to contribute to skeletal muscle atrophy [8]. ROS were initially considered a toxic byproduct of aerobic metabolism. Extensive studies have demonstrated the diverse roles of ROS in the living system. For example, ROS are involved in cell signaling in animal and plant systems; they induce cell death or necrosis and regulate gene expression [9, 10]. Hydrogen peroxide produced during ROS generation stimulates ubiquitin conjugation to muscle proteins and increases the expression of E3 ligases (Atrogin-1 and MuRF1). This process indicates that ROS catalyzes muscle proteins and increases muscle loss [11]. ROS-related gene expression is known to be increased by skeletal muscle atrophy and is inhibited by antioxidants [12]. Therefore, materials with antioxidant activity have potential use in therapeutic intervention for skeletal muscle atrophy.

Glucocorticoids (GC) represent some of the most common therapeutic compounds used to treat inflammation. Exogenous GCs are administered to treat inflammatory diseases, such as rheumatoid arthritis, and asthma. However, high dosage and long-term use of GCs in catabolic diseases, such as cancer cachexia, starvation, and sepsis, result in side effects, such as loss of muscle mass and function [13].

GCs are well-known to induce skeletal muscle atrophy by directly affecting muscle proteins and increasing the transcription of the ubiquitin-proteasome pathway components when administered at high doses [14]. Dexamethasone (DEX), a synthetic GC, is thus used to induce experimental muscle atrophy, as proven by many previous studies that DEX inhibits protein synthesis and stimulates protein degradation [15]. Further, in DEX-induced muscle atrophy, fast-twitch glycolytic muscle fibers (type 2B) are more affected than other fiber types (slow-twitch oxidative muscle fibers, type I) [16].

*Valeriana fauriei* (VF) is a medicinal plant used for treating depression, insomnia, anxiety, and cardiovascular diseases and is widely found in China, Japan, and South Korea [17, 18]. VF possesses antioxidant activity and contains 150–200 bioactive chemicals, including monoterpenes, alkaloids, and iridoids [19–21]. We have previously reported that VF alleviates hepatic steatosis in obese mice by inducing autophagy [22]. VF also exerts neuroprotective effects and reduces pain and stress [23]. However, the effect of VF on myotube differentiation and muscle atrophy has not been investigated.

In the present study, we examined whether VF could inhibit DEX-induced muscle atrophy in C2C12 myotubes and C57BL/6 mice. We also examined the effect of VF on oxidative stress in muscle atrophy.

## 2. Materials and Methods

### 2.1. Extract Preparation

Dried VF root was obtained from herbal medicine market in Korea. We stored the dried VF root at 4°C until use. The pulverized powder of VF root was extracted at 80°C for 2 h with 70% ethanol to extract both the polar and nonpolar components. The extract was filtered, concentrated using a rotary vacuum evaporator (Büchi Labortechnik AG, Flawil, Switzerland), freeze-dried, and stored at -20°C until further use.

### 2.2. DPPH Scavenging

Scavenging activity for 1,1-diphenyl-2-picrylhydrazyl (DPPH), a stable free radical, was measured spectrophotometrically. For the DPPH assay, the VF extract was dissolved in absolute ethanol at varying concentrations (0.5, 1, 2.5, 5, and 10 *μ*g/mL) and mixed with an equal volume of DPPH solution (0.2 mM). The mixtures were mixed vigorously and incubated at room temperature for 30 min in the dark. Absorbance was measured at 514 nm using a Tecan microplate reader (Tecan, Männedorf, Switzerland). The percentage of DPPH radical scavenging was calculated as follows.

(1) $$\begin{array}{c} \text{DPPH radical scavenging }\left(\%\right)=\frac{\left(A_{\text{control}}-A_{\text{sample}}\right)}{A_{\text{control}}}\times 100 \end{array}$$

### 2.3. ABTS Scavenging

The 2,2′-azino-bis(3-ethylbenzothiazoline-6-sulfonic acid) (ABTS) radical was produced by reacting ABTS aqueous solution (7.4 mM) with an equal volume of potassium persulfate (2.6 mM) for 12–16 h at room temperature in the dark. The produced ABTS radical solution was diluted with PBS; then, the optimal absorbance was 0.7 at 734 nm. The VF extract was dissolved in dimethyl sulfoxide (DMSO) at varying concentrations (0.5, 1, 2.5, 5, and 10 *μ*g/mL). For the ABTS assay, 198 *μ*L of ABTS radical solution and 2 *μ*L of VF extract were mixed and incubated for 2 h at room temperature. Absorbance was measured at 734 nm using a Tecan microplate reader (Tecan). The percentage of ABTS scavenging was calculated as per the following formula.

(2) $$\begin{array}{c} \text{ABTS radical scavenging }\left(\%\right)=\frac{\left(A_{\text{control}}-A_{\text{sample}}\right)}{A_{\text{control}}}\times 100 \end{array}$$

### 2.4. Cell Lines and Culture Conditions

C2C12 myoblast cells (CRL-1772) were purchased from American Type Culture Collection (ATCC, Manassas, VA, USA). C2C12 cell was cultured as previously described [24, 25]. Briefly, the cells were cultured in Dulbecco's modified Eagle's medium (DMEM, Hyclone Co., Logan, UT, USA) containing 10% fetal bovine serum (FBS, Hyclone) and antibiotics (100 U/mL penicillin and 100 *μ*g/mL streptomycin, Invitrogen, Carlsbad, CA, USA) at 37°C in a 5% CO_2_ humidified incubator. When the cells reached 100% confluence, differentiation was induced by changing the medium to DMEM containing 2% horse serum (Gibco, Invitrogen Inc., Carlsbad, CA, USA) and antibiotics. The medium was then renewed every 2 days. After 4 days of differentiation, 5 *μ*M DEX (D4902, Sigma-Aldrich, St. Louis, Missouri, USA) was added and incubated for 24 h to induce myotube atrophy.

### 2.5. Cytotoxicity Assay

The cytotoxicity of the VF extract was measured using the MTT (3-(4, 5)-dimethylthiazol−2-y1)-2, 5-diphenyltetrazolium bromide) assay. C2C12 cells were seeded in a 96-well culture plate at 1 × 10^4^ cells/well and incubated for 24 h at 37°C with 5% CO_2_. Then, the cells were treated with various concentrations of VF extract, from 0.5 *μ*g/mL to 10 *μ*g/mL, or with didrovaltrate (DI) at 0.125 *μ*M to 10 *μ*M, for 24 h. DMSO was used as the vehicle. Following VF exposure, 20 *μ*L of 5 mg/mL MTT reagent in phosphate-buffered saline (PBS) was added to each well and incubated for 4 h at 37°C with 5% CO_2_. Then, the solution was removed from all wells, and 200 *μ*L DMSO was added to solubilize the produced formazan. Absorbance was measured at 570 nm using a Tecan microplate reader (Tecan).

### 2.6. Luciferase Reporter Assay

ERR*γ* and PPAR*δ* overexpressing cells were produced as previously described and treated with VF [24]. After 24 h incubation, the luciferase activity was measured using the Dual-luciferase reporter assays kit (Promega, Madison, Wisconsin, USA) with Tecan microplate reader (Tecan) following the manufacturer's description.

### 2.7. DCF-DA Staining

Intracellular ROS levels were measured by DCF-DA staining. DCF-DA (nonfluorescent) interacts with intracellular ROS and generates 2,7-dichlorofluorescein (DCF, fluorescent). C2C12 cells (2 × 10^5^ cells/well) were seeded into a 6-well culture plate and cultured for 24 h at 37°C with 5% CO_2_, followed by treatment with the VF extract or DI for a further 24 h at 37°C with 5% CO_2_. In the last half hour, the cells were incubated with 2.5 *μ*M DCF-DA (Sigma-Aldrich) at 37°C and washed twice with PBS. Fluorescence intensity was detected at excitation and emission wavelengths of 480 nm and 530 nm, respectively, using a Tecan microplate reader (Tecan). The images of stained cells were acquired using a microscope (IX71, Olympus, Tokyo, Japan).

### 2.8. Immunofluorescence Staining and Fusion Index (%)

The differentiation efficiency of C2C12 was evaluated by total myosin heavy chain (MHC) immunofluorescence as previously described [24]. After differentiation, C2C12 cells were washed twice with PBS, fixed with 4% para-formaldehyde for 30 min, permeabilized with 0.05% saponin for 30 min, and blocked with 1% bovine serum albumin (BSA) for 1 h at room temperature. The cells were then incubated overnight with the primary antibody against total MHC (MF20, Developmental Studies Hybridoma Bank, IA, USA), in 1% BSA at 4°C, followed by incubation with the secondary antibody, goat anti-mouse IgG (H + L) (DyLight 488) (#4408, Cell Signaling Technology, Beverly, MA, USA) in 1% BSA, for 30 min at 27°C. After MHC staining, the cell nuclei were counterstained with DAPI for 1 min at 27°C. Images were obtained using a microscope (IX71, Olympus), image analysis was performed using ImageJ software (NIH, Bethesda, MD, USA), and the fusion index was calculated as per the following formula:

(3) $$\begin{array}{c} \text{Fusion index }\left(\%\right)=\frac{\text{Number of nuclei in myotube}}{\text{Total number of nuclei}}\times 100 \end{array}$$

### 2.9. Western Blotting

Whole-cell lysates and quadriceps muscle tissue proteins were extracted using RIPA buffer (89901, Thermo Fisher Scientific, Waltham, USA) containing a protease inhibitor cocktail (#5871, Cell Signaling Technology). Nuclear and cytoplasmic proteins were extracted using NE-PER Nuclear and Cytoplasmic Extraction Reagents Kit (78833, Thermo Fisher Scientific) following the manufacturer's protocol. Protein concentrations were measured using the Pierce BCA Protein Assay Kit (23227, Thermo Fisher Scientific); equal amounts of protein were mixed with the sample buffer and heated at 95°C for 5 min. The proteins were separated using sodium dodecyl sulfate-polyacrylamide gel electrophoresis and transferred onto a polyvinylidenedifluoride membrane (162-0177, Bio-Rad). The membrane was blocked with 5% skim milk in TBST at room temperature for 1 h and incubated with a primary antibody, diluted in TBST, at 4°C for 16 h. After washing with TBST, the membrane was incubated with a horseradish peroxidase-conjugated secondary antibody, diluted in TBST, at room temperature for 1 h. After washing with TBST, protein expression was detected using the Pierce ECL Western Blotting Substrate kit (32106, Thermo Fisher Scientific). The primary antibodies used are listed in Table 1.

### 2.10. Real-Time Quantitative Reverse Transcription-Polymerase Chain Reaction (qRT-PCR)

Total RNA from C2C12 cell lysates was extracted using NucleoSpin RNA plus (MN740984.250, Macherey-Nagel, Düren, Germany). Total RNA from skeletal muscles was extracted using the RNeasy Fibrous Tissue Mini Kit (74704, Qiagen, Hilden, Germany). cDNA was synthesized using the ReverTra Ace qPCR RT Master Mix (TOFSQ-20, Toyobo, Osaka, Japan), on a Bio-Rad thermal cycler (Bio-Rad, Hercules, USA). cDNA was amplified using the ViiA7 system (Applied Biosystems, Foster City, CA) following SYBR green qPCR amplification under the following conditions: 95°C for 1 min and 40 cycles of 95°C for 15 s and 60°C for 1 min. Relative mRNA expression levels were calculated using the 2^−*ΔΔ*Ct^ method and were normalized to 18S rRNA. The primer sequences used are listed in Table 2.

### 2.11. Animal Experiments

**Figure 1:**
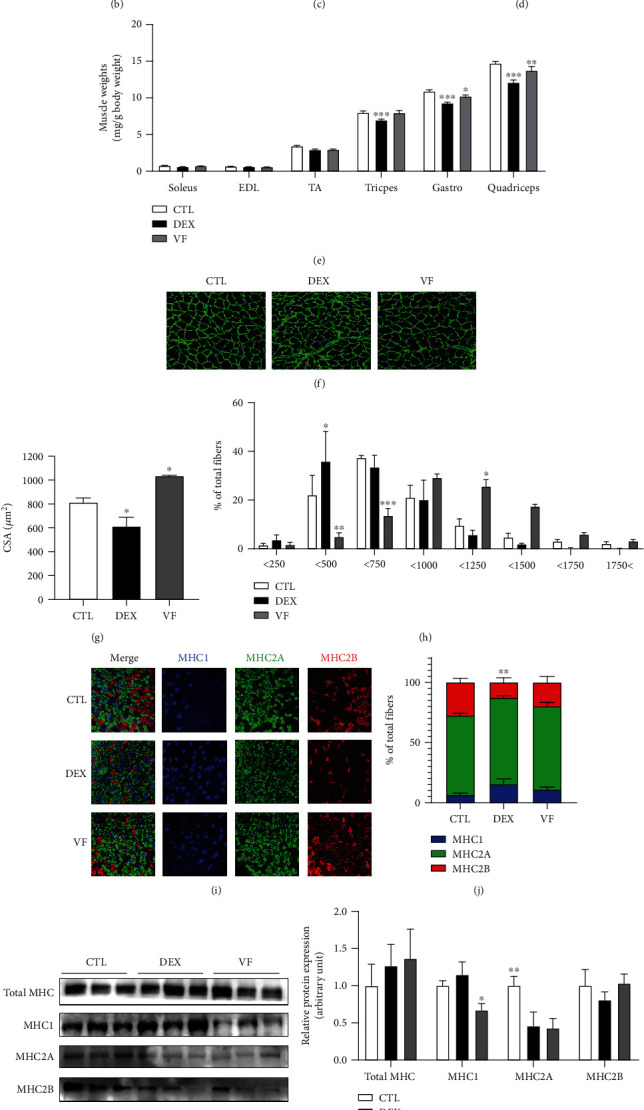
VF alleviates DEX-induced muscle atrophy in C57BL/6 mice. (a) Scheme of the experiments conducted in mice. (b–d) Exercise capacities were measured by running distance (meters) (b), running time (minutes) (c), and grip strength (grams) (d). (e) Skeletal muscle weights (mg) were normalized to body weights (g). (f) Myofiber cross-sectional area (CSA, *μ*m^2^) of gastrocnemius muscles was measured by immunohistochemical staining for laminin. Representative images of laminin staining in gastrocnemius muscle. (g) Mean CSA (*μ*m^2^) value for gastrocnemius muscles. (h) Distribution of gastrocnemius muscle CSA. (i) Immunohistochemical staining of gastrocnemius muscles with triple-labeling for MHC1 (blue, Type 1 fibers), MHC2A (green, Type 2A fibers), and MHC2B (red, Type 2b fiber). (j) Ratio of MHC isoforms. (k) Protein expression of MHC isoforms (total MHC, MHC1, MHC2A, and MHC2B) as measured by western blot analysis. (l) The quantification of western blot (k) by ImageJ (*n* = 3). (m) The mRNA expression of muscle atrophy markers, *Atrogin-1*, *Murf1*, and *Mstn*, in quadriceps muscles as measured by qRT-PCR. The data are presented as the mean ± SEM. Statistical significance was determined by one-way ANOVA. ^∗^*P* < 0.05, ^∗∗^*P* < 0.01 versus the CTL group.

Seven-week-old male C57BL/6 mice (*n* = 30) were purchased from Orient Bio Inc. (Seongnam, Korea) and housed at 21–23°C with a 12 h light-dark cycle. After one week of acclimation, the mice were divided into three groups (*n* = 10/group): vehicle-control group (CTL), DEX-control group (DEX), and 0.05% VF administrated (VF) group. The mice were fed an experimental diet for 8 weeks. The diets were based on AIN-93M (Dyets, Bethlehem, PA), and VF was mixed with the powder supplemented by daily intraperitoneal injections of DEX (Handong, Seoul, Korea) at 15 mg/kg body weight for the last 18 days of the experimental period. After the experiment, the mice were euthanized with 5% isoflurane, and tissue samples were collected. All samples were stored at -80°C until used. The detailed animal experiment flowchart is shown in Figure 1(a). The animal experiments were approved by the Institutional Animal Care and Use Committee of the Korea Food Research Institute (KFRI-M-19030).

### 2.12. Treadmill

Muscle performance was evaluated after 2 days of training as previously described [25]. Running began at 10 m/min for 20 min with a 10% incline and gradually increased by 2 m/min every 2 min. When the mouse remained on the shock grid for 10 s, the mouse was considered fatigued, and the experiment was stopped. The running distance (m) and running time (min) were measured.

### 2.13. Grip Strength Test

The grip strength of fore limbs was measured using a grip strength test (Bioseb, Chaville, France). Mouse grip strength was measured five times for each animal, and the mean of five measurements was calculated. Grip strength was expressed in grams.

### 2.14. Histologic Analysis

Gastrocnemius muscles were stained as previously described [24]. Gastrocnemius muscles were immersed in Tissue-Tek Optical Cutting Temperature compound (Sakura Finetech, Tokyo, Japan) in a cryo mold and frozen in liquid nitrogen-cooled isopentane. The samples were stored at -80°C until used. The muscles were cut in the middle using a cryo-microtome (CM1850, Leica Microsystems, Wetzlar, Germany) at -20°C and sectioned at a thickness of 7 *μ*m. The muscle section was placed on a precool (-20°C) slide, fixed with 20% ice-cold acetone for 30 min at room temperature, and locked with 10% FBS in PBS for 1 h at room temperature. To analyze the cross-sectional area (CSA), tissue sections were incubated overnight at 4°C with anti-laminin conjugated with Alexa Flour 488 (NB-300, Novus Biologicals) in 1% BSA. To analyze muscle type transition, tissue sections were incubated overnight at 4°C with antibodies against MHC1 (BA-F8, DSHB), MHC2A (SC-71-C, DSHB), and MHC2B (BF-F3, DSHB) in 1% BSA. The primary antibodies were mixed and added simultaneously. The tissue sections were then incubated with the respective secondary antibodies, rabbit anti-mouse IgG2b (DyLight 405) (NBP1-72922, Novus Biologicals), Alexa Fluor 488 goat anti-mouse IgG (A21121, Invitrogen), and Alexa Fluor 594 goat anti-mouse IgM (A21044, Invitrogen) in 1% BSA at room temperature for 1 h. Following staining, tissues were washed twice with PBS and mounted with Fluoroshield (F6182, Sigma-Aldrich). Stained tissue images were acquired using a confocal microscope (ECLIPSE C1 Plus, Nikon, Tokyo, Japan).

### 2.15. Quadriceps Muscle Antioxidant Activity Measurement

Quadriceps muscle protein was homogenized in PBS and stored at -80°C for analyzing the antioxidant activities in the muscle. Protein concentrations were measured using the Pierce BCA Protein Assay Kit (23227, Thermo Fisher Scientific), and equal amounts of protein were used for measurement. Total antioxidant activity was measured using the Total antioxidant capacity assay kit (ab65329, Abcam, Cambridge, UK) following the manufacturer's protocol. SOD activity was assessed using a superoxide dismutase activity assay kit (ab65354, Abcam). Muscle glutathione, malondialdehyde, and NADPH oxidase 1 levels were determined using the glutathione (GSH) ELISA kit (MBS267424, MyBioSource, San Diego, CA, USA), malondialdehyde ELISA kit (MBS741034, MyBioSource), and NADPH oxidase 1 ELISA kit (MBS2885477, MyBioSource), respectively, following the manufacturer's protocol.

### 2.16. Statistical Analysis

Data were analyzed using the GraphPad Prism version 8.0 (GraphPad Software, Inc., La Jolla, CA) and were expressed as the means ± SD (*in vitro*) or SEM (*in vivo*). One-way ANOVA was used for statistical analyses, followed by Dunnett's multiple comparison test. A probability value of *P* < 0.05 was used as the criterion for statistical significance.

## 3. Results

### 3.1. VF Demonstrates ROS Scavenging Activity

**Figure 2:**
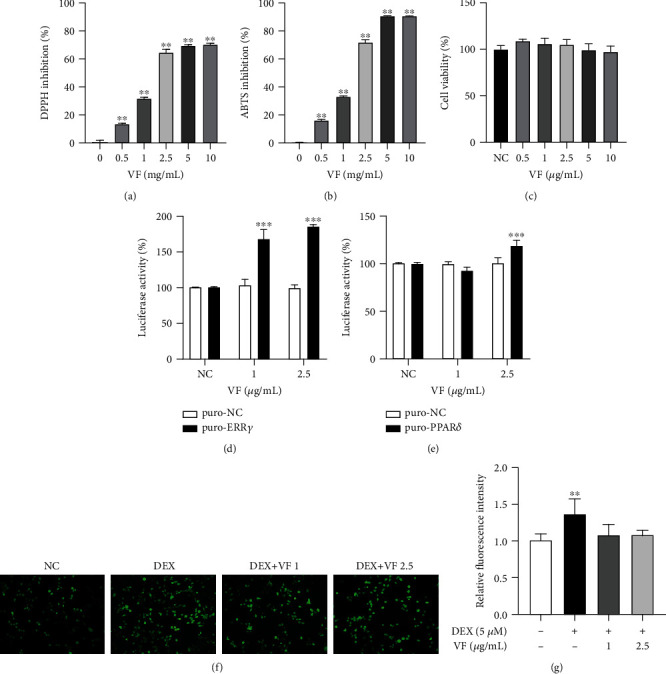
Antioxidant activity and cytotoxicity of VF extract on C2C12 myoblasts. Antioxidant assays were performed with VF extract at concentrations from 0.5 to 10 mg/mL. (a) DPPH inhibition (%) by VF. (b) ABTS inhibition (%) by VF. (c) Viability (%) of C2C12 cells was measured by MTT assay after 24 h of VF treatment. (d, e) The effect of VF on transcriptional activity of ERR*γ* and PPAR*δ*. (f) Representative images of oxidized DCF-DA in C2C12 myoblasts treated with DEX or/and VF. (g) Fluorescence intensity depicting ROS production measured by DCF fluorescence in C2C12 cells. The data are presented as the mean ± SD. Statistical significance was determined by one-way ANOVA. ^∗^*P* < 0.05, ^∗∗^*P* < 0.01 versus the nontreated group.

In this study, we evaluated the radical scavenging activity of VF using the DPPH and ABTS assays. When DPPH reacted with antioxidants, it was reduced and discolored from purple to yellow. ABTS radical was quenched when reacted with antioxidants. The DPPH and ABTS radical scavenging activities of VF represented by IC_50_ were 1.878 mg/mL and 1.693 mg/mL, respectively. Higher concentrations of VF showed better radical scavenging activity (Figures 2(a) and 2(b)). We then measured the cytotoxic effect of various concentrations of VF on C2C12 cells using the MTT assay (Figure 2(c)). No toxicity was observed in C2C12 cells treated with 0.5 *μ*g/mL to 10 *μ*g/mL of VF. To determine the effective concentration of VF, we performed a luciferase assay (Figures 2(d) and 2(e)). ERR*γ* and PPAR*δ* were transcription factors mediating mitochondrial metabolism and induced by exercise. 1 *μ*g/mL of VF increased ERR*γ* transcriptional activity, and 2.5 *μ*g/mL of VF increased ERR*γ* and PPAR*δ* transcriptional activity. We thus conducted the subsequent experiments with 1 *μ*g/mL and 2.5 *μ*g/mL of VF. An excess of GC is known to induce ROS overproduction in cells, which can be measured using the DCF-DA assay [26]. As expected, 5 *μ*M DEX treatment significantly induced ROS to 1.4-fold in C2C12 cells compared to that in the untreated control, whereas cotreatment with 1 *μ*g/mL or 2.5 *μ*g/mL of VF was found to suppress ROS (*P* < 0.05 or *P* < 0.01) (Figures 2(f) and 2(g)). These results suggest that VF possesses ROS scavenging activity and reduces DEX-induced oxidative stress in C2C12 myoblasts.

### 3.2. VF Prevents DEX-Induced Myotube Atrophy in C2C12 Myotubes

**Figure 3:**
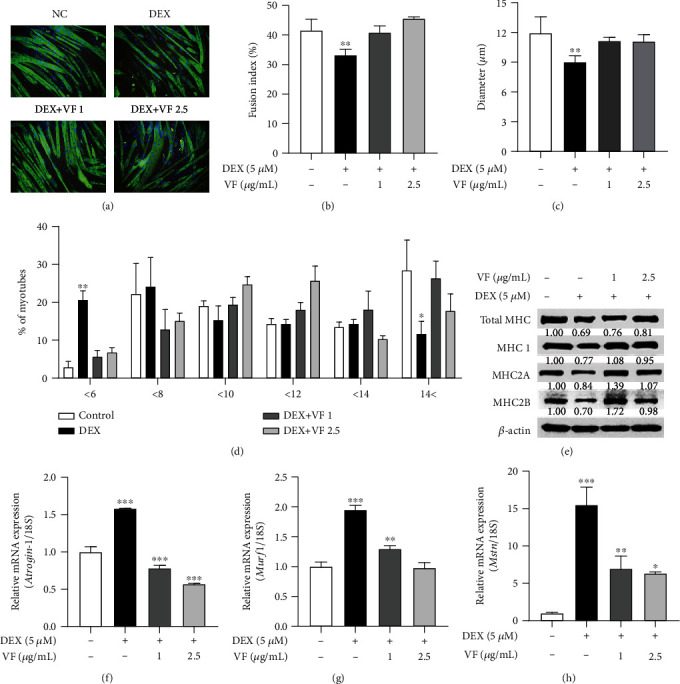
VF prevents DEX-induced myotube atrophy in C2C12 cells. (a) Immunofluorescence staining for MHC (green) and counterstaining with DAPI (blue) in C2C12 myotubes. (b) Fusion index (%) was calculated. (c) Myotube diameter (*μ*m) was measured. (d) The distribution (%) of myotube diameter (*μ*m). (e) Protein expression of MHC isoforms (total MHC, MHC1, MHC2A, and MHC2B) in VF-treated C2C12 myotubes was measured by western blot analysis. (f–h) The mRNA expression of muscle atrophy markers, including *Atrogin-1* (f), *Murf1* (g), and *Mstn* (h), was measured by qRT-PCR. The data are presented as the mean ± SD. Statistical significance was determined by one-way ANOVA. ^∗^*P* < 0.05, ^∗∗^*P* < 0.01 versus the nontreated group.

To assess the effect of VF on DEX-induced myotube atrophy, we performed immunofluorescent staining and observed the total MHC expression of myotubes (Figure 3(a)). DEX treatment dropped the fusion index level to 20%, whereas cotreatment of DEX with 1 and 2.5 *μ*g/mL of VF blocked the decrease in DEX-induced fusion index level, which then recovered to 98% and 110%, respectively, compared with nontreatment (Figure 3(b)). DEX also reduced the mean myotube diameters and increased the distribution of small myotube diameters, indicating myotube atrophy, which was effectively blocked myotube atrophy (Figures 3(c) and 3(d)). We then measured the protein expression of MHC isoforms (total MHC, MHC1, MHC2A, and MHC2B) by western blot analysis (Figure 3(e)). DEX induced a decrease in MHC protein levels, whereas VF protected DEX-evoked decrease of MHC. The mRNA expression of muscle atrophic biomarkers, such as A *trogin-1*, *Murf1*, and *Mstn*, as measured by qRT-PCR (Figures 3(f)–3(h)), was found to be upregulated by DEX and significantly decreased by VF (*P* < 0.01). These results indicate that VF prevents DEX-induced myotube atrophy in C2C12 myotubes.

### 3.3. VF Alleviates DEX-Induced Muscle Atrophy in C57BL/6 Mice

C57BL/6 mice were fed an experimental diet supplemented with 0.05% VF for 8 weeks, and muscle atrophy was induced by DEX administration in the last 18 days of the experiment (Figure 1(a)). Muscle function was evaluated by running distance, running time, and grip strength (Figures 1(b)–1(d)). The results indicated that DEX reduced muscle performance, whereas VF increased exercise ability. Measurement of muscle weights normalized to body weights (Figure 1(e)) indicated that VF prevented loss of muscle mass in DEX-induced muscle atrophy. We then performed histological analysis of the gastrocnemius muscle by laminin staining and measured the CSA (Figures 1(f) and 1(g)). DEX decreased the CSA compared with CTL, whereas VF prevented the reduction of muscle fiber size. In the muscle fiber size distribution, DEX showed the highest distribution between 200 *μ*m^2^ and 400 *μ*m^2^. However, VF showed the highest distribution between 400 *μ*m^2^ and 600 *μ*m^2^, which was higher than that with DEX (Figure 1(h)). DEX-induced muscle atrophy is known to damage MHC2B with fast-to-slow MHC type transition [16]. To measure muscle type transition, we immunohistochemically stained the MHC of gastrocnemius muscle tissue (Figure 1(i)). MHC2B was damaged by DEX administration, and the MHC2B/MHC1 ratio was decreased in DEX-treated tissues. However, VF supplementation suppressed the MHC isoform transition (Figure 1(j)). In addition, western blot analysis showed DEX-induced changes of MHC1 and MHC2 isoforms were slightly prevented by VF supplementation (Figures 1(k) and 1(l)). The mRNA expression of atrophic genes was upregulated by DEX and effectively decreased by VF (Figure 1(m)). These results indicate that VF effectively prevents DEX-induced muscle atrophy in C57BL/6 mice.

### 3.4. VF Exerts Antioxidant Activity in DEX-Induced Muscle Atrophy in C57BL/6 Mice

**Figure 4:**
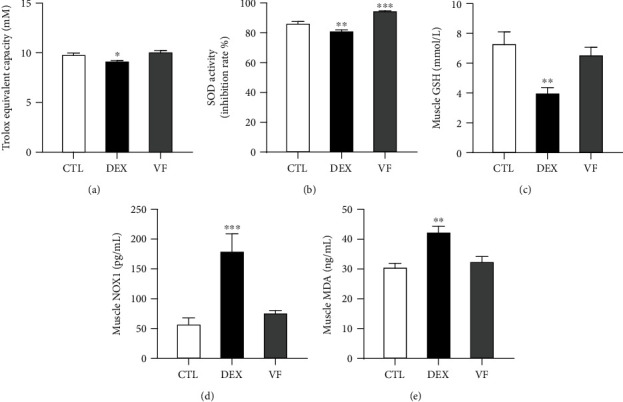
Antioxidant activity of VF in DEX-induced muscle atrophy in C57BL/6 mice. (a) Total antioxidant activity is represented by Trolox equivalent antioxidant activity. (b) SOD activity is represented by inhibition rate (%). (c) Muscle glutathione (GSH) level is presented as concentration (mmol/L). (d) Muscle NADPH oxidase 1 (NOX1) level is presented as concentration (pg/mL). (e) Muscle malondialdehyde (MDA) level is presented as concentration (ng/mL). All activities were measured in the quadriceps muscle. The data are presented as the mean ± SEM. Statistical significance was determined by one-way ANOVA. ^∗^*P* < 0.05, ^∗∗^*P* < 0.01 versus the CTL group.

ROS play an important role in muscle protein degradation during muscle atrophy, and high levels of ROS are generated in DEX-induced muscle atrophy involves a high level of ROS [27]. Thus, antioxidants have the potential to prevent muscle atrophy. We thus evaluated various markers related to ROS/antioxidants in quadriceps muscles using commercial kits. Total antioxidant activity was represented by Trolox equivalent antioxidant capacity. DEX-treated muscle tissue had lower antioxidant capacity (9.1 mM) compared to CTL (9.8 mM), whereas VF increased the antioxidant capacity to 10.1 mM (Figure 4(a)). Next, we measured the activity of SOD, one of the most important antioxidative enzymes. SOD catalyzes the dismutation of the superoxide anion (O_2_^−^) to molecular oxygen (O_2_) and hydrogen peroxide (H_2_O_2_) [28]. SOD activity was significantly decreased by DEX (81.1%), compared with the CTL (86.3%). However, VF supplementation increased the SOD activity (94.6%) (*P* < 0.01) (Figure 4(b)). Glutathione (GSH) is an endogenous antioxidant and buffers ROS levels by modulating the antioxidative cellular defense machinery. Here, we observed that DEX decreased GSH biosynthesis whereas VF could prevent GSH loss (Figure 4(c)). Figures 4(d) and 4(e) show that VF supplementation significantly prevented DEX-induced elevation of NOX1 and MDA levels. These results suggest that VF efficiently acts as an antioxidant against DEX-induced ROS in skeletal muscle tissues.

### 3.5. VF Inhibits Nuclear Translocation of GC Receptor (GR)

**Figure 5:**
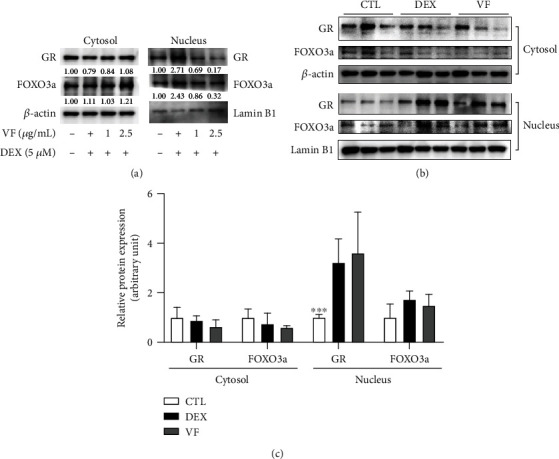
VF inhibits the nuclear translocation of the GC receptor (GR). (a) GR and FOXO3a were detected by western blot analysis in C2C12 myotubes cytosol and nucleus fractions, respectively. (b) GR and FOXO3a were detected by western blot analysis in mouse quadriceps muscle cytosol and nucleus fractions, respectively. (c) The quantification of western blot (b) by ImageJ (*n* = 3). The data are presented as the mean ± SEM. Statistical significance was determined by one-way ANOVA. ^∗^*P* < 0.05, ^∗∗^*P* < 0.01 versus the CTL group.

An excess of DEX promotes the catabolic effect of muscle and DEX-activated GR [29]. To investigate whether VF prevents GR translocation and FOXO3a activation, we performed western blot analysis in C2C12 myotubes and C57BL/6 mice. The nuclear translocation of GR by DEX was effectively decreased by VF treatment dose-dependently in C2C12 myotubes (Figure 5(a)). The inhibitory effect of VF was also observed in DEX-induced atrophic muscle tissues (Figures 5(b) and 5(c)). Subsequently, the nuclear translocation of FOXO3a by DEX was inhibited by VF in muscle cells and muscle tissues. Our results suggested that VF ameliorated muscle atrophy by inhibiting DEX-induced GR translocation from the cytosol to the nucleus and inhibiting FOXO3a activation.

### 3.6. Didrovaltrate (DI) Inhibits DEX-Induced Myotube Atrophy in C2C12 Cells

**Figure 6:**
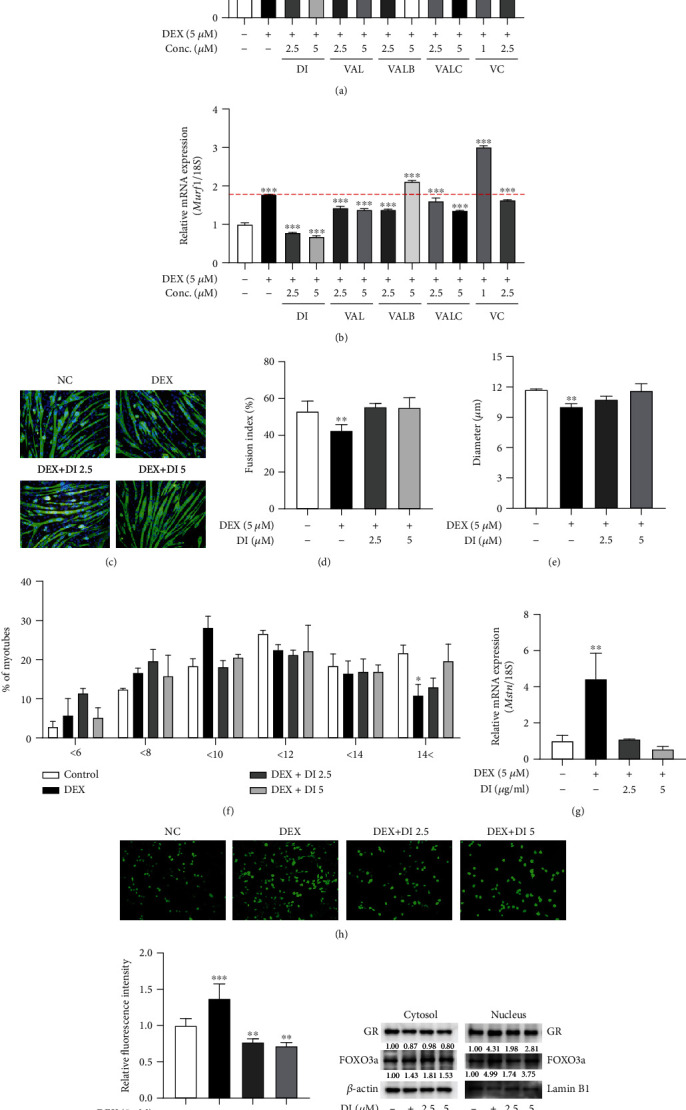
Didrovaltrate (DI) inhibits DEX-induced myotube atrophy in C2C12 cells. The inhibitory effect using iridoids of VF. (a, b) The mRNA expression of muscle atrophy markers, such as *Atrogin-1* and *Murf1*, in C2C12 myotubes. DI was found to be the most effective compound. (c) Immunofluorescence staining for MHC (green) and counterstaining with DAPI (blue) in C2C12 myotubes. (d) The fusion index (%) was calculated. (e) Myotube diameter (*μ*m) was measured. (f) The distribution (%) of myotube diameter (*μ*m). (g) The mRNA expression of *Mstn* in C2C12 myotubes. (h) Representative images of oxidized DCF-DA in C2C12 myoblasts treated with DEX or/and DI. (i) ROS production was measured by DCF fluorescence intensity in C2C12 myoblasts. (j) GR and FOXO3a were detected by western blot analysis in C2C12 myotube cytosol and nuclear fractions, respectively. The data are presented as the mean ± SEM. Statistical significance was determined by one-way ANOVA. ^∗^*P* < 0.05, ^∗∗^*P* < 0.01 versus the nontreated group.

In a previous study, we reported five iridoids, namely, didrovaltrate (DI), valtrate (VAL), valeriotriate B (VAL B), valeriotetrate C (VAL C), and valechlorine (VC), as functional components of VF [22]. To test the effects of iridoids on DEX-induced myotube atrophy, we measured the mRNA expression of *Atrogin-1* and *Murf1* (Figures 6(a) and 6(b)). As a result, the 2.5 *μ*M and 5 *μ*M DI and 2.5 *μ*M VAL B inhibited upregulation of *Atrogin-1* and 2.5 *μ*M and 5 *μ*M DI, VAL, and VAL C, and 2.5 *μ*M VAL B inhibited the increase of *Murf1*. Of the five iridoid compounds tested, DI showed the most effective inhibitory effects. Therefore, we selected DI as a bioactive compound of VF and conducted subsequent experiments with a concentration of 2.5 and 5 *μ*M. To determine the effect of DI on myotube differentiation, we measured the fusion index and found that DI effectively attenuated DEX-induced myotube atrophy and increased the fusion index to 104% (Figures 6(c) and 6(d)). DEX treatment dropped the fusion index level to 80% whereas cotreatment of DEX with 2.5 and 5 *μ*M of DI blocked the decrease in fusion index level. Instead, 2.5 and 5 *μ*M DI treatment showed a higher fusion index level than without DEX treatment. DEX caused the decrease in myotube diameters and the number of myotubes larger than 14 *μ*m and DI significantly blocked the myotube atrophy (Figures 3(c) and 3(d)). As shown in Figure 6(g), *Mstn*, a muscle atrophic marker, was also suppressed by DI. Following these results, we hypothesized that DI might attenuate DEX-induced muscle atrophy by ROS scavenging and inhibition of GR translocation. As expected, DI inhibited DEX-induced oxidative stress in C2C12 myoblasts, as confirmed by a DCF-DA assay (Figures 6(h) and 6(i)). We also demonstrated that DI effectively inhibited nuclear translocation of GR in C2C12 myotubes (Figure 6(j)). Taken together, DI was found to inhibit muscle atrophy through ROS scavenging and to inhibit GR translocation, suggesting that DI is the bioactive compound of VF for the prevention of muscle atrophy.

## 4. Discussion

The loss of muscle mass and function is defined as muscle atrophy, and increasing protein degradation is one of the reasons for muscle atrophy. Oxidative stress is caused by the imbalance between the production of ROS and antioxidant capacity, and this leads to mitochondrial dysfunction, protein damage, increased ubiquitin-proteasome activity, and reduced protein synthesis [30]. ROS level is increased during muscle atrophy, and reducing ROS could be a way of preventing muscle atrophy. VF has higher ROS scavenging activity compared to *Valeriana officinalis*, which belongs to the same plant genus [20, 27]. We also reported that VF moderated hepatic steatosis by lipophagy [22]. In the present study, we demonstrated that antioxidant activity of VF attenuated DEX-induced muscle atrophy and that DI was a functional component of VF.

*Valeriana fauriei* contains various flavonoids that have antioxidant capacities, such as hesperidin, apigenin, quercetin, and kaemferol, which reduce ROS levels [31]. The antioxidant activity of *Valeriana fauriei* plays a role in preventing disease, aging, and cancer and inhibiting DNA damage [20]. We also demonstrated the DPPH and ABTS inhibitory activities of VF. VF decreased DEX-induced ROS production in C2C12 cells.

DEX, a synthetic GC, is used as a muscle atrophy inducer. DEX-induced muscle atrophy results from decreasing fusion index and myotube diameters, increasing expression of atrophic markers, such as Atrogin-1 and MuRF1, and MHC protein degradation [6, 7]. We demonstrated that VF downregulated Atrogin-1 and MuRF1 and consequently prevented DEX-induced myotube atrophy. These results showed that ROS reduction is effective in preventing muscle atrophy.

The muscle UPS regulates muscle proteostasis and an increase of muscle-specific E3 ligases, such as Atrogin-1 and MuRF1, are related to muscle atrophy. DEX administration in mice increased E3 ligases, resulting in reduced muscle mass, strength, and muscle area [32]. Overexpression of Atrogin-1 in myotubes induces atrophy, and mice lacking MuRF1 are protected from DEX-induced muscle atrophy [33]. MSTN is also a negative regulator of skeletal muscle mass that regulates muscle fiber number and limits muscle fiber growth [34]. MSTN was upregulated by DEX administration to rats and was suppressed by RU486, which is a DEX antagonist [15]. We confirmed that DEX administration induced the loss of muscle mass and function and upregulation of *Atrogin-1*, *Murf1*, and *MSTN*. Further, VF supplementation effectively suppressed muscle atrophy-related genes. DEX is known to induce not only upregulation of atrogenes but also fast-to-slow muscle type transition. MHC2B has high susceptibility to DEX-induced muscle atrophy because of the lower level of PGC-1*α* than MHC1 and MHC2A. Conversely, high levels of PGC-1*α* are highly resistant to atrophy [35, 36]. As a result, the exposure to DEX induced the transitions in MHC isoforms, from fast to slow type muscle. In the present study, VF supplementation alleviated the DEX-evoked muscle fiber type transition in C57BL/6 mice.

Suppressing ROS generation and increasing antioxidant enzymes are described as an approach for alleviating muscle atrophy. ROS is generated by exercise in skeletal muscle, and as a defensive response, antioxidant enzymes such as SOD and GSH are induced [37]. SOD, which is an antioxidant enzyme, was reduced by DEX treatment and oxidative stress markers, namely, GSH and MDA, increased and decreased, respectively, by ROS formation [27, 38]. NAPDH oxidases (NOX) are reported as major sources of ROS. Among them, NOX1 is upregulated by DEX in smooth and skeletal muscles. Therefore, NOX1 was used as a ROS marker for diagnosing DEX-induced diseases [39]. We demonstrated that DEX reduced antioxidant enzymes, and VF supplementation prevented the loss of antioxidant enzymes in the quadriceps muscle. Therefore, reducing ROS is one of the effective methods of preventing DEX-induced muscle atrophy.

DEX exerts biological effects through GR, a member of the nuclear receptor family. In skeletal muscle, GR participates in protein degradation control via transcription factor regulation and mediates the effects of GC [40]. The loss of fast-type muscle, a key phenotype of DEX-induced muscle atrophy, is related to the aforementioned PGC1*α* level as well as the GR expression. GR has high expression in fast-type muscle [41]. GR is primarily expressed in the cytoplasm, and upon binding GC, it translocates into the nucleus. Translocated GR activates forkhead transcription factors (FOXO). FOXO1, FOXO3a, and FOXO4 are key regulators of muscle atrophy, and these activate Atrogin-1 and MuRF1 [42]. We showed that DEX-induced translocation of GR as well as FOXO3a from the cytoplasm to the nucleus. However, VF prevented GR translocation and FOXO3a activation in C2C12 myotubes and C57BL/6 mice. These results showed that VF inhibited DEX-induced muscle atrophy by preventing DEX/GR/FOXO3a/Atrogin-1 and MuRF1 cascade.

*Valeriana fauriei* mainly contains iridoids, which is a type of monoterpenoid. Iridoids have various biological activities, such as anti-inflammatory, antidepression, and antioxidant. We previously reported that VF contains five iridoids, namely, VC, VAL, VAL C, VAL B, and DI, and alleviates hepatic steatosis by lipophagy [22]. Among iridoids, DI is classified in the valepotrate group and acts as a musculotropic agent due to its influence on calcium influx and binding in the muscle [43]. We showed that DI has antioxidant activity and inhibited DEX-evoked myotube atrophy and FOXO3a activation [22]. Therefore, we considered that DI is the functional compound of VF that prevents DEX-induced muscle atrophy. However, it needs to be confirmed in an *in vivo* experiment.

## 5. Conclusions

In conclusion, we demonstrated that VF prevents DEX-induced muscle atrophy and GR translocation in C2C12 myotubes and C57BL/6 mice. VF downregulated muscle atrophic genes and suppressed DEX-evoked MHC type transition. VF also decreased the ROS levels in atrophied muscles. DI, an active compound of VF, have ROS scavenging activity and inhibited DEX-induced muscle atrophy. Therefore, we suggested that VF has beneficial effects as a therapeutic supplement for muscle atrophy, and these results provide a basis of clinical trials.

## Figures and Tables

**Table 1:** Primary antibodies used for western blot analysis.

Antigen	Host	Dilution	Provider Cat no.
Total MHC	Mouse	1 : 1000	DSHB MF-20
MHC 1	Mouse	1 : 1000	DSHB BA-F8
MHC 2A	Rabbit	1 : 1000	Abcam ab91506
MHC 2B	Mouse	1 : 200	DSHB BF-F3
Beta-actin	Mouse	1 : 5000	Santa Cruz SC-47778
Glucocorticoid receptor	Rabbit	1 : 1000	Cell signaling #3660
FOXO3a	Rabbit	1 : 1000	Cell signaling #2497
Lamin B	Mouse	1 : 1000	Invitrogen MA1-06104

**Table 2:** Primer sequences used for qRT-PCR.

Gene	Forward	Reverse
*Atrogin-1*	GACTGGACTTCTCGACTGCC	TCAGGGATGTGAGCTGTGAC
*Murf1*	GCTGGTGGAAAACATCATTGACAT	CATCGGGTGGCTGCCTTT
*Mstn*	ACGCTACCACGGAAACAATC	GGAGTCTTGACGGGTCTGAG
*18S*	CTCAACACGGGAAACCTCAC	CGCTCCACCAACTAAGAACG

## Data Availability

The data used to support the findings of this study are available from the corresponding author upon request.
